# Lemierre Syndrome Associated with *Streptococcus constellatus* and Atypical Vascular Involvement: A Case Report and Review of the Literature

**DOI:** 10.3390/idr16060086

**Published:** 2024-11-12

**Authors:** Luca Pipitò, Antonio Anastasia, Fabrizio Passalacqua, Giulio D’Agati, Floriana Di Figlia, Benedetta Romanin, Silvia Bonura, Raffaella Rubino, Agostino Inzerillo, Caterina Sarno, Antonio Cascio

**Affiliations:** 1 Department of Health Promotion, Mother and Child Care, Internal Medicine and Medical Specialties “G D’Alessandro”, University of Palermo, 90133 Palermo, Italy; luca.pipito@community.unipa.it (L.P.); floriana.difiglia@community.unipa.it (F.D.F.); 2 Infectious and Tropical Disease Unit, AOU Policlinico “P. Giaccone”, 90127 Palermo, Italybenedetta.romanin@policlinico.pa.it (B.R.);; 3 Department of Radiological Sciences, AOU Policlinico “P. Giaccone”, 90127 Palermo, Italy

**Keywords:** *Streptococcus constellatus*, Lemierre syndrome, thromboses, internal jugular thrombosis, transverse sinus thrombosis

## Abstract

**Background:** Lemierre syndrome is a rare and life-threatening disease. It is characterized by septic thrombophlebitis of the internal jugular vein, historically associated with *Fusobacterium necrophorum* infection. However, atypical cases and associations with other organisms have been reported. **Methods:** Here, we describe a challenging case of Lemierre syndrome in a 71-year-old woman caused by *Streptococcus constellatus* and review the related literature. **Case:** The patient experienced multiple hospital admissions due to misdiagnoses and developed thrombosis involving the internal jugular vein and transverse sinus bilaterally, pulmonary complications including the formation of a pseudoaneurysm, and occipital abscess. She presented with headaches, neck pain, and blindness. Prolonged antibiotic therapy was administered, leading to gradual improvement of symptoms, with partial resolution of blindness. Prophylaxis with intramuscular penicillin was prescribed at discharge. **Conclusions:** Our case underscores the importance of considering Lemierre syndrome in patients who present with multiple thrombotic events affecting the intracranial circulation and/or jugular veins, particularly in those already receiving anticoagulation therapy or with no identifiable cause for thrombosis, even in the absence of sore throat or fever.

## 1. Introduction

Lemierre syndrome, also known as postanginal sepsis or necrobacillosis, is a rare and life-threatening condition typically following an infection in the head and neck region, most commonly pharyngitis. It is characterized by septic thrombophlebitis of the internal jugular vein, historically associated with *Fusobacterium necrophorum* infection [1,2,3,4]. A rise in the incidence of reported cases has been described over the past 30 years, with rates possibly reaching up to 5.5 cases per million in 2017 [4]. Atypical presentations and variants have been reported, and other bacteria have also been linked to the syndrome, such as members of the *Streptococcus viridans* group, *Enterobacteriaceae*, *Eikenella corrodens*, *Bacteroides*, *Streptococcus pyogenes*, and *Staphylococcus aureus* [1,5]. The infection can spread from the jugular vein, causing septic emboli in distant organs, leading to a wide range of severe complications and symptoms [1,2,3,4]. While the exact pathogenesis is not fully understood, it is believed that bacteria from the initial infection in the head and neck trigger thrombophlebitis in local veins, especially the internal jugular vein, which then leads to the spread of distant metastatic septic emboli [3]. Early clinical manifestations may include systemic signs and symptoms such as fever and rigors or local ones such as neck pain, with more severe symptoms arising from septic emboli leading to metastatic complications [1,2,3,4]. Diagnosis is often challenging due to the variability in presentation and the lack of a standardized definition, and then cases can be easily missed. Additionally, the inappropriate duration of antimicrobial treatment can increase the risk of relapse and further complications. Although *Streptococcus constellatus* has been identified as a rare causative agent in Lemierre syndrome, only a few cases have been reported in the literature [6,7,8,9,10,11,12,13,14]. In this report, we present a rare case of Lemierre syndrome caused by *Streptococcus constellatus*, characterized by neuro-ophthalmological and pulmonary involvement, with multiple relapses due to initial misdiagnosis. We also review previously documented cases from the literature.

## 2. Case Description

### 2.1. First Admission to the Hospital

A 71-year-old woman presented to an emergency department in March because of sharp pain in the right orbital region, ptosis, and headache. She was admitted to a neurology unit. Ten days prior, she had experienced self-limiting right-sided otalgia without fever. Her medical history was notable for paroxysmal atrial fibrillation, diagnosed the previous year and managed with rivaroxaban, as well as bacterial pneumonia that occurred 16 months before the current presentation. She also had a history of hypertension and dyslipidemia. There was no history of dental infections or dental interventions in the last years. A computed tomography (CT) angiography performed during that admission revealed thrombosis of the right superior ophthalmic vein. Additionally, cerebral magnetic resonance imaging (MRI) with contrast performed after four days identified thrombi in the left transverse sinus, sigmoid sinus, and internal jugular vein. The MRI also showed phlebitis of the right and left superior ophthalmic veins and mild left-sided meningeal enhancement. She was treated with linezolid and ceftriaxone empirically for ten days, suspecting meningitis, along with heparin and corticosteroids for venous thrombosis and possible vasculitis. No spinal puncture and blood cultures were performed. Screening for thrombophilia was negative, including Factor II and Factor V mutations, MTHFR (A1298C mutation), and autoimmune disorders. Transthoracic echocardiography showed no evidence of infective endocarditis, and a follow-up MRI two weeks later showed partial recanalization of both ophthalmic veins with no signs of phlebitis, though the other thrombi persisted. The patient improved clinically and was discharged on acenocoumarin therapy.

### 2.2. Second Admission to the Same Hospital

**Figure 1:**
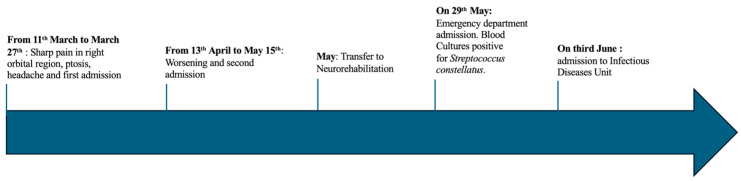
Timeline of patient’s clinical history.

In April, the patient’s condition deteriorated, leading to a fall with a mild head injury. She was readmitted, and a CT scan revealed new ischemic lesions in the cerebellum and periventricular regions. Due to worsening clinical symptoms, including fever, psychomotor slowing, and disorientation, treatment with linezolid, acyclovir, meropenem, and dexamethasone was initiated for suspected meningoencephalitis. During her hospital stay, the patient experienced bilateral visual loss. Additionally, an MRI revealed extensive recent bilateral occipital ischemic lesions, a left cerebellar lesion, and multiple areas of pathological enhancement, raising suspicion of inflammatory meningitis. A spinal puncture was not performed due to the risk of bleeding, and blood cultures were not reported in the patient’s medical records. A total-body CT scan did not reveal any neoplastic lesions. After clinical stabilization and partial recovery of visual acuity, the patient was transferred to a neurorehabilitation center in May. However, a few days later, she again experienced headaches and rigors, prompting her to transfer to an emergency department. A CT scan revealed a new nodular lesion (4 cm in diameter) with central cavitation (2.5 cm in diameter) in the left upper pulmonary lobe, along with bilateral pleural effusion. Cerebral MRI with contrast showed a worsening of the pre-existing venous thromboses involving the left transverse sinus, sigmoid sinus, and internal jugular vein. Blood tests were notable for elevated C-reactive protein (CRP) and leukocytosis. Blood cultures were taken and were positive for *Streptococcus constellatus*. The patient was then transferred to our infectious diseases unit with a suspected diagnosis of Lemierre syndrome in June. A timeline of the patient’s history is depicted in Figure 1.

### 2.3. Admission to the Infectious Diseases Unit of Our University Hospital

On admission, the patient presented complaining of cough, left-sided neck pain, malaise, and blindness. At this point, the patient’s history was checked again, and it was confirmed that there was no history of recreational drug use. Her vital signs were as follows: pulse rate of 78 beats per minute, respiratory rate of 24 breaths per minute, blood pressure 170/70 mmHg, and oxygen saturation of 97% on room air. Physical examination revealed partial impairment of spatial–temporal orientation, blindness, and left lower limb weakness, rendering her unable to maintain an upright or seated position. Blood tests showed elevated CRP levels at 143 mg/L, anemia with hemoglobin at 7.6 g/dL, red blood cell count of 2.59 × 10^6^ cells/μL, leukopenia with a white blood cell (WBC) count of 4870 cells/μL (neutrophils 87.7%), and thrombocytopenia with platelet (PLT) count of 75,000/μL. Additionally, total cholesterol was 273 mg/dL, triglycerides were 491 mg/dL, and lactate was elevated at 2.6 mmol/L (Table 1).

**Figure 2:**
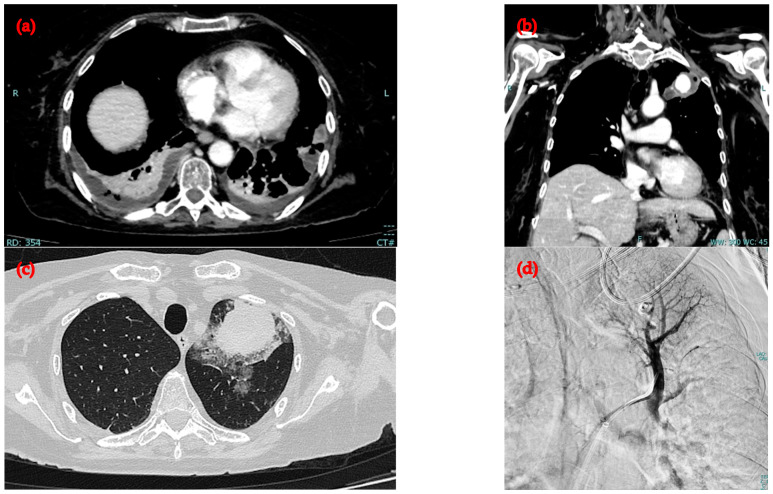
The CT images with contrast show the following: (**a**) a basal right pleural effusion with consolidation and multiple air bubbles within; (**b**) in the apico-dorsal segment of the left upper lobe, an inflammatory lesion containing an intralesional pseudoaneurysm of the apico-dorsal segmental branch of the left upper lobe bronchial artery (maximum diameter 2.3 cm); (**c**) alveolar hemorrhagic involvement following the rupture of the pseudoaneurysm; (**d**) embolization procedure through the placement of 5 coils.

Screening for methicillin-resistant *Staphylococcus aureus* (MRSA) on a pharyngeal swab was positive. Three sets of blood cultures, using bottles for both aerobic and anaerobic microorganisms, confirmed the isolation of *Streptococcus constellatus*. Therapy with teicoplanin and piperacillin-tazobactam was initiated. Pulmonary tuberculosis was excluded via negative sputum tests and Quantiferon TB Gold assay. Screening for the JAK2 V617F mutation was negative. Antibiogram results confirmed sensitivity to penicillin, and transthoracic echocardiography showed no evidence of infective endocarditis. As a result, teicoplanin was discontinued, and piperacillin-tazobactam (4.5 g every 6 h) was continued, followed by de-escalation to ceftriaxone (2 g daily) and oral metronidazole (500 mg three times daily). Therapeutic anticoagulation with enoxaparin 4000 iu twice daily was administered. CRP levels continued to rise for the first four days, reaching 264 mg/L, but subsequently showed a gradual reduction. The patient’s clinical course was complicated by abscess formation within the cavitated pulmonary nodular lesion, a pseudoaneurysm of the segmental branch of the superior bronchial artery inside the cavitation, right basal pleural empyema, and the development of new multiple pulmonary abscesses (up to 2.5 cm in diameter), as shown on chest CT (Figure 2). The pseudoaneurysm led to acute hemoptysis, necessitating urgent endovascular embolization.

**Figure 3:**
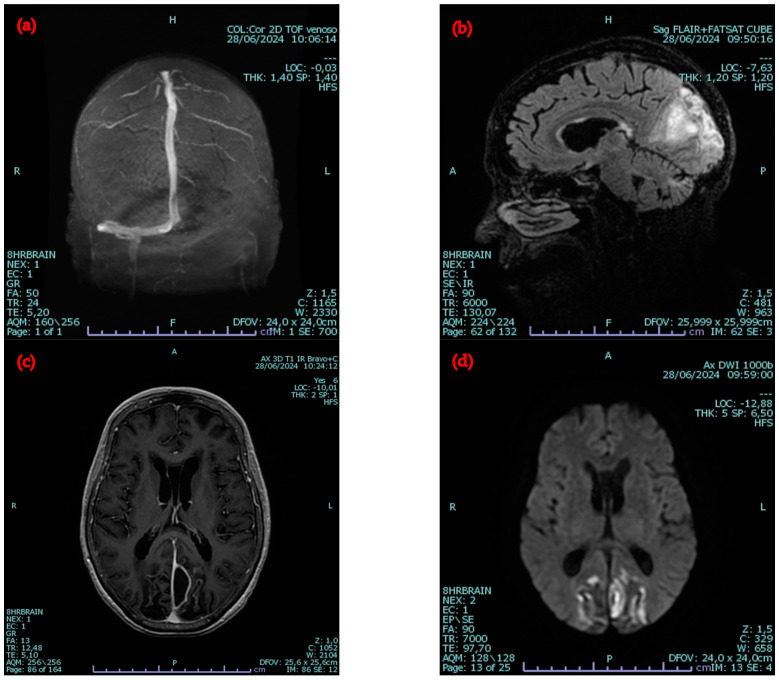
The MRI images show the following: (**a**) absence of flow signal in the left transverse sinus, the sigmoid sinus, and the left jugular vein in the intracranial segment, indicating thrombosis; (**b**) in the occipital region bilaterally, areas of altered signal compatible with sub-acute ischemic events; (**c**,**d**) an ovoid-shaped area with a maximum diameter of 28 mm, showing heterogeneous signal restriction on DWI and peripheral enhancement after contrast administration, suggestive of an abscess formation.

A neck and brain CT scan showed the left complete thrombosis of the transverse sinus with minimal filiform opacification in its proximal tract, of the sigmoid sinus, and of the most proximal portion of the internal jugular vein. On the right side, the scan revealed partial thrombosis of the transverse sinus, sigmoid sinus, and the proximal portion of the internal jugular vein. Additionally, there was partial thrombosis at the distal end of the superior sagittal sinus, along with an opacification defect indicating near-complete thrombosis of the right external jugular vein throughout its entire course. Brain MRI also showed a brain abscess near the right straight sinus on the left side (Figure 3). 

**Figure 4:**
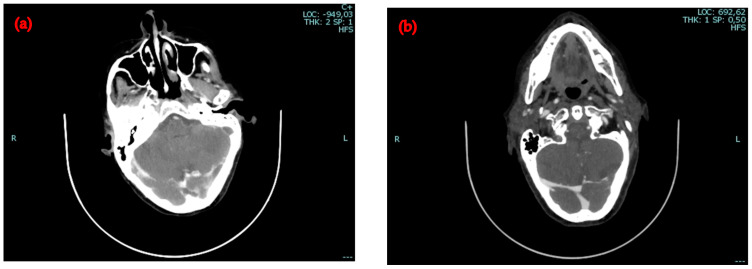
Comparison between brain CT scans with contrast on admission and discharge: (**a**) complete thrombosis of the left transverse sinus and partial thrombosis of the right transverse sinus; (**b**) resolution of the partial thrombosis of the right transverse sinus, while on the left side, the thrombo-embolic opacification defect persists, affecting the transverse sinus, the sigmoid sinus, and the proximal portion of the internal jugular vein.

The patient demonstrated a slow but progressive improvement in response to antibiotic therapy. Inflammatory markers normalized after seven weeks of antimicrobial treatment. The patient’s cognitive status and spatial–temporal orientation returned to baseline, her motor function improved, and she regained the ability to remain seated. Vision partially improved, allowing her to recognize shapes, objects, people, and colors. Ophthalmologic examinations throughout her treatment did not reveal any structural abnormalities of the eyes. A follow-up neck and chest CT performed one month later showed resolution of the partial thrombosis in the right transverse sinus, sigmoid sinus, and proximal internal jugular vein, though other thrombi persisted (Figure 4). The size of the pulmonary abscesses had decreased considerably. 

After 61 days of antibiotic therapy, the patient was transferred to a neurorehabilitation unit. Before discharge, intramuscular benzylpenicillin prophylaxis was initiated, and the first dose of 2.4 million IU was administered. A long-term prophylactic regimen was established, involving 1.2 million IU of benzylpenicillin monthly. The patient remains under follow-up at our clinic. At the follow-up examination performed one month after discharge, the patient was in good general condition and afebrile, with no neck pain or other complaints. She had recovered her vision except for the peripheral visual fields. Blood tests were normal, and CRP was 3 mg/L.

## 3. Materials and Methods

In addition to the case described in this article, we conducted a literature search on Lemierre syndrome associated with *Streptococcus constellatus*, using only the PubMed database. We employed the search strings “Lemierre syndrome” AND “Streptococcus constellatus” and “Streptococcus constellatus” AND “Thrombosis”. No language restriction was applied during the research. We yielded 22 relevant articles.

## 4. Discussion

### 4.1. Lemierre Syndrome Definition

Classic Lemierre syndrome is typically diagnosed based on the presence of an oropharyngeal infection and subsequent thrombosis or septic embolism involving the internal jugular vein, usually caused by *Fusobacterium* species. However, atypical and variant forms of Lemierre syndrome have been described in the literature, characterized by multiple venous thromboses and various primary infection sites [2,5]. According to recent definitions supported by case reports and substantial consensus, Lemierre syndrome can now be broadly defined as a bacterial thrombophlebitis associated with a range of primary infections caused by various microbial agents. In fact, variant forms of Lemierre syndrome may present with infections originating outside the oropharynx, and they may not involve the internal jugular vein or *Fusobacterium necrophorum* [2,5]. Among these causative agents is *Streptococcus constellatus*. Only a few cases of typical and variant Lemierre syndrome associated with *Streptococcus constellatus* have been reported in the literature [6,7,8,9,10,11,12,13,14,15,16,17,18,19,20,21,22,23,24,25,26,27]. Consistent with our case, the largest analysis of Lemierre syndrome, which included data from 712 patients, demonstrated that atypical Lemierre syndrome is more common in older individuals. It is characterized by the absence of *Fusobacterium* spp. infection, involvement of veins other than the internal jugular vein, and the lack of a typical primary infection site, such as pharyngitis [28]. The study found that *Streptococcus* spp. and *Staphylococcus* spp. were the most frequently isolated bacteria when *Fusobacterium* spp. was not detected. However, the specific types of *Streptococci* involved were not identified [28]. In our case, the laboratory results were consistent with a previous review that reported laboratory findings from a large number of Lemierre syndrome cases [29]. This review showed that the median CRP and platelet counts were 122 mg/L and 61,000/μL, respectively. Our patient’s WBC count was lower than the median reported value of 17,000 cells/μL [29].

We conducted a review of the existing literature about *Streptococcus constellatus* and Lemierre syndrome. Nine cases were initially diagnosed as Lemierre syndrome [6,7,8,9,10,11,12,13,14], while 13 others were not identified as Lemierre syndrome. They presented with *Streptococcus constellatus* infection, intracranial or ophthalmic venous system thrombophlebitis, and a suspected primary infection in the head or neck region [15,16,17,18,19,20,21,22,23,24,25,26,27].

### 4.2. Primary Infection

Our case of Lemierre syndrome differs in several aspects from the typical presentation of this syndrome. First, the infection occurred in an elderly woman, whereas Lemierre syndrome usually affects younger individuals. However, previous cases of *Streptococcus constellatus*-associated Lemierre syndrome have also described older patients, with a median age of 46 years (interquartile range 23–57), with 68% of patients aged 38 years or older [6,7,8,10,11,13,14,17,18,20,21,22,24,25,26,27]. Our case can be considered atypical due to the absence of a clear oropharyngeal infection, though the patient did report self-resolving right otalgia without fever before presentation, which could indicate an infection involving the head or neck region. Among the cases reported in the literature, the primary infection site was unknown in three cases [10,11,18]. In the others, primary infections involved the teeth in six cases [6,7,13,17,21,24], the paranasal sinuses in eight [9,15,19,20,22,23,26,27], the pharynx in three [12,16,25], and the ear in one case [8]. No full-text report was available for one case [14].

### 4.3. Clinical Picture

Fever was common and was reported in 77% of cases; however, in our patient, fever was not observed during hospitalization in the infectious diseases unit, likely due to prior steroid therapy administered for suspected meningoencephalitis and vasculitis. The clinical presentation of Lemierre syndrome can be highly variable. Our patient presented primarily with neuro-ophthalmological symptoms, including headache, sharp pain in the right orbital region, and ptosis. Other reported cases in the literature describe symptoms such as headache [6,8,9,10,15,17,19,20,25,26,27], facial swelling and pain [7,19,21,25,27], ophthalmic symptoms [6,7,17,19,20,21,24,25,27], oropharyngeal complaints [10,12,16], gastrointestinal symptoms [12,15,17,27], neurological signs [16,22,23], and flu-like symptoms [14,27]. Shoulder pain has also been reported [10]. Ocular findings in these cases included blepharoptosis, conjunctival chemosis, blurred vision, diplopia, ophthalmoplegia, periorbital and orbital edema, erythema, and pain. As in our patient, neck pain or stiffness was common in many cases [6,8,9,10,13,18,23].

### 4.4. Venous Thromboses and Other Complications

In our patient, thrombosis of the left internal jugular vein was detected following the thrombosis of the left superior ophthalmic vein. It is noteworthy that *Streptococcus constellatus* has been shown to exhibit thrombin-like activity, which may explain the extensive and severe venous thrombosis [30]. Other reported cases of Lemierre syndrome also showed extensive venous thrombosis. The internal jugular vein was involved in 32% of cases [8,9,10,12,19,24,26], while 86% of cases reported thrombosis in other cerebral or ocular veins [6,7,8,9,13,15,16,17,18,19,20,21,22,23,24,25,26,27]. The most frequently affected veins were the cavernous sinus (53%) and ophthalmic veins (31%). Other affected veins included the transverse, sigmoid, petrosal, and sagittal sinuses. In our case, transverse and sigmoid sinuses and ophthalmic veins were involved. Venous thrombosis outside the head and neck region was observed in only one case, involving the left superficial femoral vein extending to the left popliteal vein [11]. Our case was further complicated by the formation of a brain abscess, ischemic lesions in the occipital cortex leading to blindness, and pulmonary involvement with a pseudoaneurysm near a cavitary lung lesion. Pulmonary lesions were reported in 50% of previous cases [6,7,8,9,10,11,12,13,21,24,25], while brain involvement was seen in 54% of cases [8,9,15,16,19,20,22,23,24,25,26,27]. Brain complications included abscess formation, acute infarction, meningitis, and empyema. Additionally, cranial nerve palsy was reported in four cases [7,16,17,23]. Abscesses involving the skin, pharynx, muscles, and other structures were also reported in 36% of cases [6,10,13,15,16,19,21,24]. Finally, our case is notable for the presence of a pulmonary artery pseudoaneurysm. The largest analysis of arterial complications in Lemierre syndrome highlighted that severe arterial complications, including mycotic aneurysms, may occur in a minority of patients. However, these complications typically involve the internal carotid artery [31].

### 4.5. Differential Diagnoses

In our case, the diagnosis of Lemierre syndrome was delayed, leading to a prolonged and complicated clinical course with multiple septic complications and recurrent hospitalizations. The rarity of such cases, combined with the atypical presentation and the patient’s advanced age, contributed to the diagnostic challenge. Differential diagnoses included meningoencephalitis, tuberculosis, right-sided endocarditis, and thrombophilic syndromes. The pulmonary involvement with a cavitary lesion and a pseudoaneurysm could have been mistaken for a Rasmussen’s aneurysm, suggesting pulmonary tuberculosis. The formation of a pseudoaneurysm near the cavitary lung lesion required urgent embolization. Multiple venous thromboses and pulmonary lesions could be associated with right-sided endocarditis, which was repeatedly excluded. Thrombophilic syndromes were also considered due to the patient’s extensive venous thromboses despite anticoagulant therapy, which was initiated due to a history of atrial fibrillation. The use of anticoagulants in Lemierre syndrome remains debated [1,2,3]. In 68% of previously reported cases of Lemierre syndrome caused by *Streptococcus constellatus*, both heparin and oral anticoagulants were administered [8,9,10,11,13,14,15,16,17,18,19,21,22,24,26,27]. An analysis of a large retrospective cohort of patients with Lemierre syndrome showed significantly reduced morbidity and mortality in those treated with both antibiotics and anticoagulants [32]. Our case demonstrated that thromboses can persist despite anticoagulation and broad-spectrum antibiotic therapy, confirming previous observations and underscoring the importance of early diagnosis, timely initiation of treatment, and intensive monitoring [28]. Further studies are required to better understand the role of anticoagulant therapy. The cerebral venous thrombosis and abscesses were likely a result of retrograde extension from the internal jugular vein thrombosis. Finally, the isolation of *Fusobacterium necrophorum* should not be considered essential for diagnosis, as this may result in significant diagnostic delays, as previously noted by Hill et al. [33]. Moreover, *Fusobacterium necrophorum* may not always be detected, or other organisms may be isolated, as seen in our case.

### 4.6. Treatment

Initially, suboptimal antibiotic treatment during the first two hospitalizations led to only partial improvement. Prolonged antibiotic therapy was necessary for a gradual recovery, including partial restoration of vision. Limited antibiotic penetration into fibrin clots and abscesses may have contributed to this delayed response. However, some authors suggest that anticoagulation therapy can shorten the duration of antibiotic treatment by dissolving the clot and exposing bacteria trapped in the thrombus [5]. The duration of treatment in other cases ranged from 4 weeks [13,15] to 6 months [8,9], with anticoagulation therapy typically continued until thrombosis resolution. Surgical intervention was necessary in 50% of cases [7,10,11,13,15,16,19,23,24,26,27]. The outcome was generally favorable, with full recovery reported in 73% of cases [6,8,9,10,11,12,13,15,16,17,18,19,20,21,22,26], although one case reported a less favorable outcome [23]. For our patient, we decided to start monthly intramuscular penicillin prophylaxis to prevent relapse, considering the extensive venous involvement. At the one-month follow-up visit, the patient was apyretic and reported no fever, headache, or neck pain. She had experienced a resolution of blindness, with only a residual deficit in the peripheral visual field. To the best of our knowledge, no other cases have been reported using prophylactic treatment in Lemierre syndrome. However, we believe that prophylaxis may benefit our patient until the resolution of the venous thrombi. The patient is currently under follow-up and is scheduled for a repeat MRI for further evaluation. A limitation of our study is that the previous hospitalizations occurred at other institutions in Sicily, and the timeline before our admission was reconstructed based on the patient’s documentation and family history. According to the patient’s records, microbiological tests, including blood cultures, were not conducted during the first two hospitalizations. Additionally, endocarditis was not definitively ruled out, as only transthoracic echocardiography was performed.

## 5. Conclusions

Our case underscores the importance of considering Lemierre syndrome in patients who present with multiple thrombotic events affecting the intracranial circulation and/or jugular veins, as well as neck stiffness, particularly in those already receiving anticoagulation therapy or with no identifiable cause for thrombosis, even in the absence of sore throat or fever. Neck pain and stiffness are common symptoms that might be misinterpreted as meningitis. Before starting antibiotic therapy, early blood cultures should be obtained to identify pathogens associated with Lemierre syndrome.

This condition may be underrecognized, as Lemierre syndrome is often considered a “forgotten” disease. Cases like ours are crucial for raising awareness about this condition. Prolonged antibiotic therapy is recommended to prevent recurrence, and prophylactic strategies should be considered in complex cases with frequent recurrences.

## Figures and Tables

**Table 1:** Patient’s examinations upon admission to the infectious diseases unit.

Laboratory Analysis	Patient’s Result	Reference Range
WBC (cells/μL)	4870	4000–11,000
Neutrophils (cells/μL)	4270	4000–7400
Lymphocytes (cells/μL)	520	2000–4800
Monocytes (cells/μL)	70	160–1000
PLT (n/μL)	75,000	150,000–450,000
Hb (g/dL)	7.6	12–16
ALP (U/L)	98	35–104
ALT (U/L)	21	0–35
AST (U/L)	8	0–35
GGT (U/L)	43	5–36
Bilirubin (mg/dL)	0.31	<1.2
Creatinine (mg/dL)	0.38	0.51–0.95
CRP (mg/L)	143	<5
Total cholesterol (mg/dL)	273	<200
Triglycerides (mg/dL)	491	<150
Fibrinogen (mg/dL)	1032	150–450

WBC: white blood count, PLT: platelets, ALP: alkaline phosphatase, ALT: alanine transaminase, AST: aspartate aminotransferase, GGT: gamma-glutamyl transferase, CRP: C-reactive protein.

## Data Availability

No further data are available regarding the clinical case described.
